# Reviews of Books

**Published:** 1923-06

**Authors:** 


### REVIEWS OF BOOKS.

### THE MEDICAL SIDE OF THE WAR.

**History of the Great War: Medical Services; Hygiene of the War.** (His Majesty's Stationery Office; 2 vols. £1 1s. net per volume.)

These volumes are deeply interesting, not only to all concerned with public-health, but to a much wider public. They are a record of strenuous work done by thousands of medical men and their assistants to maintain the general health of the millions who went to war, and to ward off from them the attacks of specific diseases. From the arctic to the equator, and far down south from that, this strenuous work was done and carried on right round the world. Every infectious disease came within the scope of the investigations, and at least one formerly unknown was discovered and tracked to its cause.

The provision of habitations for every climate, and for the most varied conditions of season, weather and stringency of war, was planned and carried out with wonderful success. Comfort and good health were secured in circumstances in which hope of either seemed vain. Clothing had to be modified, improved and devised for zero temperatures and for tropic heat, for drenching downpours and continuous drought, for levels below the surface of the sea, and for heights above mountain tops. Good measure of success cheered these efforts, and the chapters on housing and clothing are as interesting as any in these two volumes, of which every page presents something worth the reading.

The section dealing with footgear is instructive. It could usefully be read by the general public. No one who has examined many pairs of British feet can have failed to note the crippling and deformity bad boots have produced. Few men, still fewer women, have feet the shape Nature meant or are without callosities and exostoses of an unsightly and troublesome nature. If half the thought were given in civil life, in days of peace, that is given by army authorities in times of war to the proper fitting of the boot to the foot, and the shaping of the extremities to a perverted idea of a beautiful boot abandoned, much of national inefficiency for war would be obviated, and a lumber of men useless in that they cannot march would no longer be a weakness to States in arms. The mothers of the civilised world have it in their power to remove this handicap, for the most part of the deformation comes of bad shoeing of children. The vanities of youth do the rest.

Populations dwelling in highly developed countries are so accustomed to conveniences for water supply and sewage disposal that few ever think about the subject or imagine the difficulties such problems present when a vast assemblage is collected in camp or a force has to be billetted on the habitations of more primitive people. The pages dealing with latrine accommodation in Russia are amazing. The wonder grows that Russians, or any other people, can endure to live under such conditions. The book is a full manual of the hygiene of war in any and every part of the world. No matter where a force, in the future, may be sent, in these two volumes a man may find practically all that is necessary to be known by those responsible for the food, clothing and housing in the field of white troops in that locality. Here may be learnt the best methods of disposal of all forms of refuse and excreta ; in wet lands or dry lands, in flat lands or mountainous, in arctic or tropic ; and details of improvisations of sorts and kinds, from dwellings and kitchens to destructors and fly traps. The strategy and tactics of the fights to repel the attacks of special diseases are set forth in full detail. Especially interesting are the chapters that deal with parasites of the human body and diseases and troubles caused by them. Minutely particular researches were made into the life-habits of many lowly creatures whose existence is a danger to human life. New facts were discovered, old statements were confirmed or refuted, and ingenious measures concerted to destroy the bringers and hosts of disease. Other creatures were called in to aid imperial man ; even ducks were enlisted to guard him against bilharziasis. More than in the common understanding of the term was the Great War a World War.

How great was the success of science in preventing disease and lessening the attrition of sickness will never be known. It can be estimated to an extent by considering the medical history of wars of the past. To go back no further, in the South African War, in aggregate, some half million men served from first to last. Of them ten per cent. fell out from typhoid fever, and of those attacked sixteen per cent. died. In the Great War, ten times as many British troops took the field, yet only one-eighth as many cases occurred in all that vast number, and the fatal cases were some thirty times fewer. Admirable are the triumphs of preventitive medicine ! All the patient research work, the laborious experiment, the thorough and thoughtful organisation and administration recorded in these volumes was marvellously successful and extremely creditable to the men and women engaged. They were set to maintain the fighting efficiency of armies, to repel disease or to cure it, and to keep at the highest possible the forces in the field. They succeeded in their task to immeasurably higher degree that ever before. Great was the saving of life, great the boon to humanity the result of their labours.

And yet this thought intrudes—was there saving, was there boon ? Had it not been for these men's work would not the armed nations in the field, weakened by the decimations of disease, have sooner fallen out of grips, have stood back one from another appalled by the horrid ravage of loathsome afflictions, been unable to smite at one another, and, therefore, much earlier than was the case, have come to pause and made a peace before there had been half of the slaughter that culled of the best or a tithe of the loss of wealth that is hampering this generation of Europe and will hamper generations to come ? Duty was done, success was achieved, but was a boon given ? Are there to-day one score fewer of men dead than would have been dead had scientific medicine not upheld the forces in the field ?

### THE VITAMINS.

**Vital Factors of Foods: Vitamins and Nutrition.** By Carleton Ellis, S.B., F.C.S., and Annie Louise Macleod, Ph.D. (Chapman & Hall; 25s. net.)

Ever since Forster began to experiment with synthetic foods in the middle of last century it has been known that, in addition to the primary constituents, proteins, fats and carbohydrates, foods contain certain secondary, but unknown substances which are essential to the well-being of the organism ; without these substances the body did not thrive, grow, or maintain itself in that well-balanced condition known as good health. But it was not until 1912 that Casimir Funk isolated from unpolished rice a substance which he called *vitamin*. Since that time an enormous amount of experimental research work has been performed by scores of people in order to bring to light the nature and constitution of these accessory bodies. So large a number of reports could not be obtained by everybody, nor read by a great number of people, because they are in different languages. This book, however, has been written to bring into one volume a succint account of all the work which has been done up to date, to place before the reader an epitome of the reports, and to give a clear and definite account of the vitamins, what they are, what they do in the body, and what their absence or deficiency means to us.

The authors are to be congratulated on the excellent manner in which they have done this. In the twenty chapters comprising the book they have dealt with the elementary principles of nutrition ; what the vitamins are, how they act in the body, their distribution in our foods, the effects on the vitamins of cooking, drying, canning and otherwise toring foods, and their chemical structure and properties. We learn that " The existence of three well-defined vitamins, growth-producing A, anti-neuritic B, and anti-scorbutic C, is apparently well established." Some evidence is given of the possibility of a fourth vitamin, which is closely associated with B, and is essential for growth. " A and B are both required for growth and maintenance of the body." " The most obvious symptoms of deficiency of A shown by young animals are lack of growth, diminished appetite, decline and wasting of the tissues, weariness, and lack of resistance to infection and disease." A quotation given from McCarrison about vitamin B states that " The absence of certain accessory food factors, termed anti-neuritic, leads not only to functional and degenerative changes in the central nervous system, but to similar changes in every organ of the body . . . . due to chronic inanition, derangement of the functions of the organs of digestion and assimilation, functional disturbances of the glands of internal secretion and mal-nutrition of the nervous system." Another quotation from Madsen " suggests that lack of vitamins is a factor in the causation of anæmia, chlorosis, neurasthenia, and vaso-motor disturbances."

The well-known symptoms of scurvy are associated with a deficiency of C. Due prominence is given to the work of many investigators who have shown that calcium metabolism, phosphorus metabolism, bone development, and the growth of the teeth, are under the influence of this vitamin ; that when it is absent from the food or deficient in quantity the teeth are defective, the bones deficient in calcium, and if adults are deprived of this vitamin all the symptoms of scurvy develop. The suggestion that vitamins act as hormones which excite or arouse functional activity in the organism is discussed. A whole chapter is devoted to the chemical structure and properties of vitamins, and is decidedly illuminating. Nevertheless, the conclusion is drawn that " At present there is little or no knowledge of the actual chemical nature of vitamins," which proves that, in spite of the extraction of many substances from foodstuffs which show the presence of vitamins in them by their physiological effects, it has not been possible yet to arrive at an exact knowledge of these elusive substances.

The discussion of the causation of rickets, scurvy, pellagra, and beri-beri is carried out with candour ; the experimental work thereon by various investigators is put forward in a clear and able manner. An entire chapter is devoted to the vitamins in milk, and another to vitamins for the baby. What to eat from a vitamin standpoint forms the subject of another excellent chapter. There is an appendix showing the distribution of vitamins in various foods, and the authority from which the statement is drawn. The index of authors whose works have been used in the compilation of this book is a valuable contribution to the bibliography of vitamins, and should be useful to those who desire to make a further study of the subject.

### "SOCIAL SCIENCE."

**Social Life and the Crowd.** By J. Lionel Tayler. (Leonard Parsons.)

The tone, or perhaps we ought to say the spirit, in which Mr. Tayler writes of " Social Life and the Crowd " is quite adequately set forth in part of one of his own sentences : " The first essential in modern political inquiry is the abandonment of the assumption that human beings are dominantly reasonable." We think that he is too ready to believe that the remark is novel. We hear a good deal of some modern school, or schools, of " psychologists " and what not, who have discovered that man is a complex and obscure animal.

Mr. Tayler is no believer in democracy. But, then, neither was Joseph de Maistre, who said that the people was always a child, always an imbecile, and always " absent "—never, that is to say, at hand to manage its affairs. Many others have been of that opinion. The great Savoyard would have agreed with Mr. Tayler so far. They would have differed on the question whether it is possible for any body of students to provide a remedy by study. Maistre would have said no, because, so he laid it down, nothing that has been done in politics with the deliberate intention to make a constitution, or found an institution for a definite purpose, has ever been known to answer its avowed purpose. Whatever lives and is fruitful in the life of a people has had an origin too remote and too mysterious to allow of discovery by us, and has workings so diverse and indirect that no wisdom can foresee them.

This is, no doubt, a somewhat depressing view to be taken by a speculator on political principles. It is not therefore false. Mr. Tayler is not very far from being of the same opinion, to judge by what he has to say of " complexes " and wills and instincts. He very rightly declines to be hoodwinked by names, and will not allow that any aristocracy or democracy has ever been known; and he is right if by aristocracy we are bound to mean the government of the most moral and the wisest, and if by democracy we must imply the direct administration of its affairs by a whole people acting as a body and without intermediary agents. But if aristocracy has meant the rule of certain stocks of men, families, clans, who in the modern phrase are " noble," and lead by right of their " nobility," there have been aristocracies, and even are. If " democracy " signifies the direct rule of that part of a community which has the franchise over personally free but politically unqualified persons and slaves, then there have been many, and they have administered directly in " agora " or " forum," town hall and market place. Greek cities, Rome, Venice, with its Gran Consiglio, and the Transvaal of Mr. Krüger's days with its " old burghers " and " out burghers," were democracies—in the original sense.

A Greek would have denied that our modern democracies deserve the name. They are ochlocracies, governments of the multitude, during an election by universal suffrage, and in the intervals they are oligarchies. They may be none the worse for that if the ochlocracy chooses the right men to form the oligarchy. But will it ? That is the rub. It is also the point at which the something mystic and incalculable, the deliveries of humanity's self which have no name, come in. We fear that Mr. Tayler expects more good to come of the study of " Social Psychology " and in " Crowd Studies " than they are likely to produce. Where is the student to come from except from the " crowd " ?

### A PROVOCATIVE ANGEL.

**If Britain is to Live.** By NORMAN ANGELL. (Nisbet; 2s. 6d. net.)

Mr. Norman Angell is a deliberately provocative person, who is sometimes very sensible and sometimes exceedingly wrong-headed. The mixture is piquant, and in any case he may be depended upon always to make his readers think. The thesis of this little book is that the international system under which England obtains food and clothing is disintegrating or declining in effectiveness as the result of such obstacles as political frontiers, customs barriers, competing armaments, and the like. It is all the fault of the " Nationalist organisation " of Europe, and the only way to escape is " to find the basis on which nations can combine power to maintain some system or code under which all can live in economic and political security." Mr. Angell is careful to show that he has not written an anti-war tract; war, he thinks, is not the chief danger—" economics " form the real peril. Without economic internationalism there can, he believes, be no salvation. That is a sustainable argument, and it would clearly be obscurantist in the extreme to maintain that we shall never reach such a consummation. It is clear, however, that a number of severe surgical operations will have to be performed on human nature before it becomes practicable. Low tariffs and a wider area of Free Trade are the first stages of Mr. Angell's method of cure ; but only English people—and not all of them—believe in Free Trade. It would thus seem that an economic change of heart on the part of other nations is the first essential for a new order of things which we all desiderate as much as Mr. Angell, notwithstanding the rasping way in which he sometimes preaches his gospel. He is on surer ground here than when he is speculating upon what Germany would have done with her victory if she had won the war. Some of us think we know very well what she would have done, and how she would have done it, simply because we had seen her do it before.

### KNOWING OURSELVES.

**Our Unconscious Mind, and How to Use It.** By FREDERICK PIERCE. (Kegan Paul; 10s. 6d. net.)

Of the making of popular books on psycho-analysis there would seem to be no end, and the fact indicates a desire on the part of the public to know what this new doctrine is. Many of the recent attempts to gratify this desire have been so unsatisfactory that we welcome a book such as this, which contains an adequate and accurate account of the subject, and is written with knowledge and obvious sincerity.

The book is by an American author. Mr. Pierce begins with a brief statement of the theory of psycho-analysis which makes it clear that he has grasped the fundamental principles of psychic determinism and the derivation of human action from instinctive sources, with all that these principles imply. He then gives an interesting account of a case of hysterical fixation of an arm, illustrating the psychic mechanism involved in the process and the method of relief. This leads on to an account of the conscious, the fore-conscious, and the unconscious. Mr. Pierce finds it necessary to adopt the conception of a " secondary censor " between the conscious and the fore-conscious. We pass to a discussion of Libido, a term with which the author would appear to be dissatisfied. We share this feeling. It is a question whether the term " Conatus," in the sense in which Spinoza used it, would not be far preferable. Mr. Pierce describes the development of the child into a normal or abnormal adult, and points out the waste of energy which is occasioned by unresolved conflicts He gives a good description of " will," and points out that this much-abused word really is the equivalent of " wish-force." But he does not, perhaps, make it clear to his readers that there is no " faculty " of will. A chapter on the endocrine glands seems somewhat out of place in a book of this type. It raises difficult questions as to the psycho-physical relation.

In an excellent chapter on "Autosuggestion" it is pointed out that whether the source of the suggestion is internal or external, the process of suggestion proceeds entirely within the subject. This is, of course, Baudoins's view, and it is of great importance. There is a good chapter also on the application of psycho-analytic principles in ordinary life, especially as regards the training of children. It would be well if maternity visitors had some instruction in this matter, so that they could warn their patients of the irreparable harm which may be done by well-intentioned mishandling of children in quite early infancy. It needs to be impressed upon both parents and teachers that, although the seeds of neurosis may be planted in the first seven years of life, it is in adolescence that they have their period of greatest growth.

But psychology does not merely apply to individuals. It has its national or collective side. The author gives us thoughtful observations on the "making of a contented human group," a task which should be the single objective of politicians, and he illustrates his remarks by psychological sketches of Germany, Russia, and the United States. There is a final chapter, very "American," but highly entertaining, on the newer psychology in advertising and selling. Also there is a bibliography, divided into elementary and advanced sections. The division is somewhat arbitrary, and is open to criticism. We doubt whether the lay reader will get much profit from the study of Stoddart's "Mind and Its Disorders," and Freud's latest and best book, "Introductory Lectures on Psycho-Analysis," is not mentioned. There is no index—an omission which can only be described as criminal.

### THE TREATMENT OF ANÆMIA.

**Anæmia: Its Causes and Modern Treatment.** By A. W. Fuller, M.D.(Edin.). (H. K. Lewis & Co.; 3s. 6d. net.)

In the preface to this little book the author announces that he has not adopted the time-worn method of giving an exhaustive account of other men's labours. This is rather disappointing. The science of medicine is team work, and the man who ploughs a lonely furrow, whether he is a politician or a scientist, is liable to have a very small following unless he is a Superman. The author's opinion of his colleagues is summed up in the sentence: "The first degree of anæmia is seldom diagnosed, and hitherto has been ignored by medical men." Right or wrong, this statement is sweeping and needlessly provocative. Within the strict limits of 63 small pages a little information, some of it useful, is given about the treatment of anæmia, and the author's chief standby would seem to be hypodermic injections of iron, arsenic and strychnine in isotonic serum. With regard to dosage he is vague, and the physician anxious to adopt the author's methods would find it difficult to master its details were he to be guided only by this book. Apart from the subject of anæmia, the volume contains this general hint: "The first truth to be impressed upon the majority is the fact of their own ignorance, and that the scraps of knowledge which they have pieced together into a false picture of their condition must be consigned to oblivion." It is not a very helpful book.

### MISCELLANEOUS NOTICES.

### A Directory of Nursing Homes.

Medical and Nursing Homes. The Scientific Press, Ltd. 9d.—This is the first issue of a useful little directory which should be of value to anyone seeking information as to really trustworthy homes for paying patients. The directory covers nursing homes in the United Kingdom, and the greatest care has been taken to ensure accuracy of detail. It does not pretend as yet to be exhaustive, and the Editor, the preface states, "will welcome the co-operation of all who may be in a position to assist in promoting the usefulness of 'Medical and Nursing Homes' by providing additional particulars for the next edition."

### "Harvard Health Talks."

The Causes of Heart Failure. By Dr. W. H. Robey. Harvard University Press. 4s. 6d. net.—This small volume is one of the "Harvard Health Talks" Series, and in it the author describes the signs and symptoms of heart disease in plain and simple language. By the earlier recognition of cardiac trouble and by appropriate management thereof it is maintained that many lives may be saved, especially in childhood, when the prevention of heart affections consequent upon slight rheumatic manifestations is of great importance.

### A Social Worker's Handbook.

Liverpool Social Worker's Handbook, 1923. Gledsdale & Jennings. 2s. 6d.—The fifth edition of a valuable guide issued every year by the Liverpool Council of Voluntary Aid. It is a model handbook, its information clearly arranged and its index carefully compiled. The social activities of Liverpool are manifold and the operations of the public authorities, their extent and variety, may well—as the handbook suggests—"come as a surprise to many Liverpool citizens who have not followed closely the social legislation of the past few years.

### Sufficient and Insufficient Vaccination.

The Student's Guide to Vaccination. By W. G. Aitchison Robertson, M.D. A. & C. Black. 3s. 6d.. net.—This comprehensive little book covers in fifteen chapters the whole ground of the theory and practice of vaccination. The author rightly insists upon the importance of performing the little operation in an "efficient" manner, not only within the meaning of the Act, but also in order that the individual may be adequately protected for a certain length of time. The practitioner is warned against complying with the common request of parents to vaccinate with a single small insertion. To perform the operation in this way is regarded as "a breach of medical duty; it is really taking money under false pretences."

### Starting a Nursing Home.

How to Start and Equip a Nursing Home. By Edith Holden, R.R.C. The Scientific Press. 9d. net.—This is a thoroughly sensible and enlightened little book which should be of the utmost value to any woman who is thinking of starting a nursing home. Miss Holden, who during the war was matron of the 3rd London General Hospital, has clearly a shrewd knowledge of human nature and understands how patients can be made really happy. Referring to the Nursing Home she has herself established, "We have," she writes, "tried to get a human atmosphere about the home—a most desirable thing in dealing with people who are sick and suffering. There are few rules and regulations. Visitors are given to understand that they may come and see their friends at any time, provided their visit does not interfere with the patient's health and treatment. . . . In hospital, of course, it is impossible to have visitors at all hours of the day; and when each patient has a separate room, with a little thought and trouble any supposed 'irregularities' can be made to fit in quite easily." The pamphlet is reprinted from "The Nursing Mirror."

